# Soil Microbial Communities Adjust Thermal Traits and Carbon Allocation in Response to Climate Manipulations in Subtropical Forest and Cropland

**DOI:** 10.1111/gcb.70836

**Published:** 2026-04-04

**Authors:** Carla Cruz‐Paredes, Albert C. Brangarí, Dániel Tájmel, Lettice Hicks, Ainara Leizeaga, Menale Wondie, Hans Sandén, Johannes Rousk

**Affiliations:** ^1^ Microbial Ecology, Department of Biology Lund University Lund Sweden; ^2^ Environmental Dynamics, Department of Science and Environment Roskilde University Roskilde Denmark; ^3^ Department of Ecosystem and Landscape Dynamics Institute for Biodiversity & Ecosystem Dynamics, University of Amsterdam Amsterdam the Netherlands; ^4^ Institute for Physical Geography and Ecosystem Science Lund University Lund Sweden; ^5^ Lancaster Environmental Center Lancaster UK; ^6^ Amhara Agricultural Research Institute Bahir Dar Ethiopia; ^7^ Forest Ecology, Deparment of Ecosystem Management, Climate and Biodiversity University of Natural Resources and Life Sciences (BOKU) Vienna Austria

**Keywords:** carbon use efficiency, drought, microbial growth, microbial respiration, microbial thermal traits, warming

## Abstract

Soil microorganisms regulate carbon (C) cycling, and their growth and respiration are strongly dependent on temperature. Yet it remains unclear how warming alters microbial thermal traits, community structure, and the balance between microbial respiration and growth, particularly in subtropical ecosystems where high temperatures coincide with low soil moisture, potentially constraining microbial activity. In this study, we investigated soil microbial thermal traits for growth and respiration, and the microbial community composition in two subtropical land‐uses with contrasting microclimates: a cooler, moist pristine forest and a warmer, drier cropland. Using open top chambers (OTCs) or rain exclusion shelters over 1.5 years, we quantified how experimental warming and drought altered microbial functioning and upscaled these effects using field soil temperature and moisture records. Field warming increased the abundance of warm‐adapted bacterial and fungal taxa and led to shifts in microbial thermal trait distributions toward higher minimum temperature values for microbial growth, indicating community‐level thermal adaptation. These thermal trait adaptations resulted in a modeled 36% reduction in annual soil CO_2_ efflux in warmed plots. Overall, our results show that thermal trait adaptation, driven partly by community restructuring, buffers soil C losses under warming and may enhance soil C sequestration in subtropical ecosystems. These findings showcase the importance of integrating microbial thermal traits into soil C models to improve predictions of climate‐carbon feedbacks.

## Graphical abstract

Warming increases warm‐adapted microbial taxa and shifts microbial thermal traits toward higher minimum temperature. These adaptations reduce modeled annual soil CO_2_ emissions by 36%, indicating that microbial thermal trait integration is essential for predicting soil carbon responses to climate change.

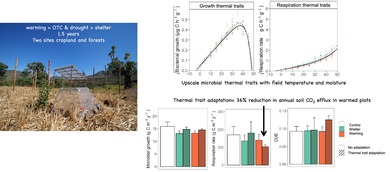

## 1 Introduction

Soils harbor more than 2500 Pg of organic carbon (C), with microbial decomposition accounting for more than half of their CO_2_ emissions (Schimel 1995). Consequently, soil microorganisms are decisive conduits in the C cycle, as they release C into the atmosphere through respiration but also store C in soil organic matter (SOM) by forming new microbial biomass that becomes necromass with long soil residence times upon death (Liang et al. 2017). Temperature is one of the key environmental drivers that regulate microbial activity in soils (Frey et al. 2013; Hagerty et al. 2014). As global temperatures continue to rise under ongoing climate change (IPCC 2021), understanding how soil microbial communities respond to temperature changes, and the extent to which they can adapt to change, is critical for predicting the impact on the C‐cycle (Bardgett et al. 2008; Crowther et al. 2016; Frey et al. 2013). Despite growing interest in microbial thermal responses, the capacity of microbial communities to adjust their functional traits to new temperature regimes, and the resulting implications for the soil C dynamics remain insufficiently understood (Bradford et al. 2016; García‐Palacios et al. 2021).

Soil warming experiments in the field have shown that warming causes a short‐term increase in soil C emissions (Melillo et al. 2002), acting as positive feedback to climate change. This initial pulse is primarily driven by the direct effect of temperature on soil microorganisms (Davidson and Janssens 2006), which accelerates metabolic rates and the decomposition of organic matter (Fang et al. 2005). However, emission rates can return to pre‐warming values within a few years, even though temperatures remain elevated (Lamb et al. 2011; Melillo et al. 2002, 2017). Two main explanations have been proposed for the gradual decline in the effect of warming on microbial process rates. First, warming depletes labile C pools, reducing substrate availability and quality (Hartley et al. 2007; Kirschbaum 2004; Melillo et al. 2002). Second, microbial communities may adapt their thermal traits to higher temperatures, influencing their functional capabilities (Bradford et al. 2010, 2019; Crowther and Bradford 2013; Dacal et al. 2019; Davidson and Janssens 2006). In addition, warming also increases evapotranspiration, reducing soil moisture, and imposing physiological stress that can constrain microbial activity and adaptation (Brangarí et al. 2021; Manzoni et al. 2012). Since soil moisture influences both substrate availability and microbial functions (Schimel 2018), its interaction with temperature is complex and often nonlinear (Cruz‐Paredes et al. 2021), making it difficult to isolate individual factor effects in field studies.

Exposure to warmer conditions can shift microbial communities and their functions, indicating that microbial communities can adapt traits to improve their performance under prevailing environmental conditions (Bradford 2013; García‐Palacios et al. 2021). Thermal trait shifts can result from species sorting or physiological adjustments, where taxa better suited to warmer conditions increase in dominance (Allison et al. 2010; Bradford et al. 2016). This has been observed in alpine, arctic, and tropical soils, where warming led to community restructuring and increased temperature minimum (*T*_min_) values, reflecting thermal trait‐adaptation to higher temperatures (Donhauser et al. 2020; Nottingham et al. 2022; Rijkers et al. 2022). This thermal trait adaptation is thought to be predominantly driven by the peak annual temperature that triggers a change in the microbial community, translating into a shift in microbial temperature relationships (Tájmel et al. 2024). However, in regions where peak temperatures coincide with extremely low soil moisture, such as subtropical dry seasons, microbial thermal trait adaptation may be constrained, as the rate of change of traits is likely proportional to microbial community turnover or metabolic rates, which may be limited by lack of moisture (Manzoni et al. 2012).

In addition to climate factors, land use is known to be a strong regulator of soil microbial communities (Delgado‐Baquerizo et al. 2018; Lauber et al. 2008), and soil temperature can also vary within the same climate zone depending on land use. For instance, forest soils are typically cooler than cropland soils due to the perennial vegetation, insulating litter layer, and canopy shading, whereas cropland soils subjected to recurrent tillage are more exposed to sunlight and weather extremes, leading to more pronounced climate effects (Brangarí et al. 2022). Different land uses also result in variations in plant communities and management practices, which affect soil structure (Sharma and Aggarwal 1984), nutrient availability, and SOM quality (Osman 2013), thereby inducing changes in microbial communities (Lauber et al. 2008) and microbial traits (Whalen et al. 2024).

Understanding the ability of microbial communities to adjust their thermal trait distributions is essential for predicting the impact on the soil C budget (Bardgett et al. 2008; Crowther et al. 2016; Frey et al. 2013). However, knowledge from recent studies showing that microbial carbon use efficiency (CUE) shifts in response to warming (Tájmel et al. 2024), and that microbial thermal traits vary geographically (Alster et al. 2023; Cruz‐Paredes et al. 2023; Karhu et al. 2014) and respond to experimental warming (Nottingham et al. 2022; Tájmel et al. 2024), has not been integrated into soil‐C models. Temperature can also indirectly influence microbial composition and resource availability (García et al. 2023; Propster et al. 2023) by reducing soil moisture (Manzoni et al. 2012), affecting plant productivity (Nottingham et al. 2015), and modifying nutrient transformation rates (Fanin et al. 2022). The uncertain outcome of the combined effects of direct and indirect temperature impacts on microbial communities (Davidson and Janssens 2006), along with the absence of a clear understanding of physiological, ecological, and functional principles (Bradford 2013; Trivedi et al. 2013; Lennon et al. 2024) reduces our ability to integrate microbial thermal trait adaptation into soil‐atmosphere C models.

To date, climate change experiments have largely focused on temperate, boreal and arctic ecosystems, leaving subtropical regions underrepresented (Cavaleri et al. 2015; Zhou et al. 2013), which is particularly problematic given their substantial soil organic C stocks (Crowther et al. 2019; Jobbágy and Jackson 2000). Given the large amount of soil C cycled in subtropical regions (Beer et al. 2010), even small changes in the soil C balance could influence the global C cycle (Cavaleri et al. 2015; Zhou et al. 2013). Despite a growing number of studies examining climate change effects in subtropical environments, most experiments still emphasize either shifts in community composition (Zhou et al. 2023; Gao et al. 2025) or changes in microbial respiration (Zhang et al. 2023), typically within a single land use type and under warming only scenarios. As a result, we still lack integrated, field‐based evidence on how simultaneous changes in temperature and moisture shape soil C cycling across subtropical landscapes, as well as how warming drives microbial thermal trait adaptation. This gap is particularly relevant because subtropical climates have pronounced dry (hot and dry) and wet (cooler and moist) seasons, offering a natural framework to disentangle the interacting effects of heat and moisture on soil microbial processes.

In this study, we investigated the distribution of microbial thermal traits in a subtropical ecosystem under two contrasting land uses, and the effects of experimental warming and drought over a period of 1.5 years on soil microbial process rates. To simulate climate change conditions, we installed rain exclusion shelters and open‐top chambers (OTCs) at two sites in Northern Ethiopia: a pristine forest and a cropland. Although both sites fall within the same climatic zone, they differ significantly in soil temperature and moisture regimes due to long‐term land use differences. We leveraged these site‐specific thermal differences to investigate whether soil microbial communities can adjust their thermal trait distributions in response to local temperature regimes and experimental treatments. Specifically, we assessed microbial thermal traits that define the temperature relationships of growth and respiration across sites and treatments to determine whether these communities exhibited local thermal trait differences. We also examined the effects of rain exclusion shelters, OTCs and land use on microbial community composition to test whether shifts in thermal trait distributions were associated with changes in community structure. Finally, to understand the consequences of these microbial responses for ecosystem C dynamics, we modeled in situ microbial growth and respiration by integrating the experimentally derived temperature response functions with field measurements of soil temperature and moisture. We hypothesized that warmer sites would host microbial communities with (i) warm‐shifted thermal trait distributions (i.e., higher *T*_min_ values) for both growth and respiration; (ii) higher representation of heat‐adapted taxa; and (iii) reduced heat‐induced soil C losses under warm temperatures, due to community trait‐driven responses that enhance growth more than respiration.

## 2 Materials and Methods

### 2.1 Study Site

**FIGURE 1:**
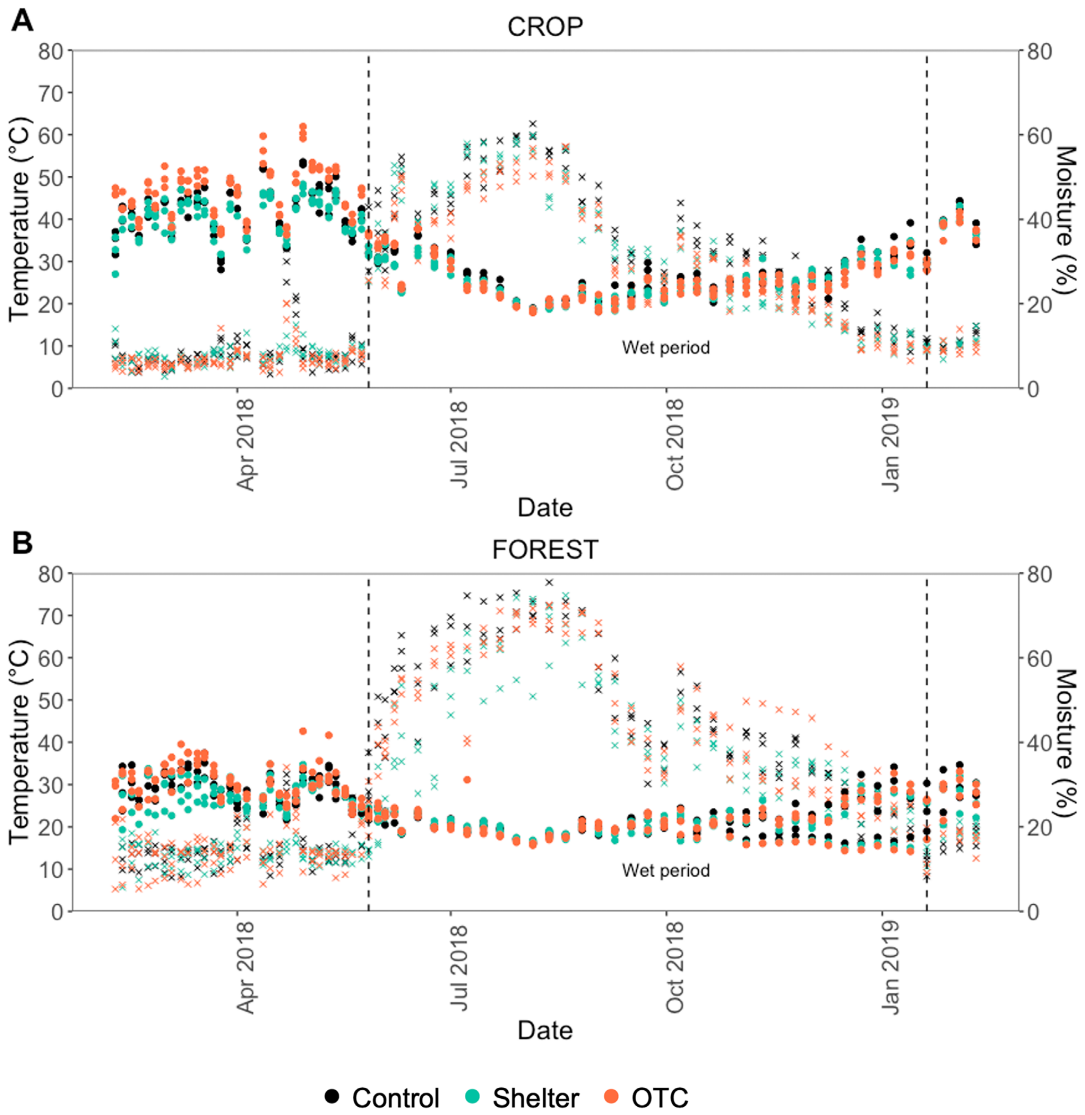
Temperature and moisture data throughout the field experiment in the two land‐uses: (A) crop and (B) forest. Different colors represent different treatments. Points represent temperature data and stars represent moisture data. Values are mean values from each plot on each day (*n* = 3).

The study sites were located near Tara Gedam in the north‐western Ethiopian Amhara region at 2110 m elevation and coordinates 12°9′42″ N, 37°44′21′E for croplands and 12°8′42″ N, 37°44′35″ E for forest sites (Assefa et al. 2017; Leizeaga et al. 2022; Hicks et al. 2025). While this region is in the subtropics, the climate of the study sites is temperate with dry winters and warm summers. The mean annual air temperature in this area is 19°C and the mean annual precipitation is 1100 mm yr.^−1^. The area exhibits a monomodal rainfall pattern, with precipitation concentrated in a single season during the year. Based on soil moisture dynamics (Figure 1), we identified a “dry season” (08/02/2018 to 26/05/2018, and 21/01/2019 to 10/02/2019) and a “wet season” (27/05/2018 to 20/01/2019). The cropland site was subjected to an annual crop rotation ( *Eragrostis tef* , *Eleusine coracana* , *Sorghum bicolor* , *Zea mays* , *Triticum aestivum* , *Guizotia abyssinica* , and *Vicia faba* ), with crops grown only during the wet season, and was ox‐ploughed between crops to a depth of 10–20 cm. The forest site consisted of a dry Afromontane remnant pristine forest composed of a mixture of indigenous tree species, including *Albizia gummifera* , *Calpurnia aurea*, *Ekebergia capensis*, *Schrebera alata*, *Rhus glutinosa*, *Afrocarpus falcatus* , *Juniperus procera*, and *Hagenia abyssinica* (Aerts et al. 2016; Zegeye et al. 2011). The soils from both sites are classified as Cambisols (FAO classification).

In November 2017, warming and rain exclusion experiments were established in both land uses, alongside control plots in a complete randomized block design. Three plots were chosen to establish warming treatments using open top chambers (OTCs) with 3 mm thick, 85 cm diameter at the top, 150 cm diameter at the base, and 35 cm tall; based on the International Tundra Experiment (ITEX) design (Marion et al. 1997). Three rain shelters (1.8 m × 1.8 m) were built at each site to simulate drought. A wooden structure with a transparent plastic cover was placed covering 80% of the area with the aim of reducing 80% of the incoming rainfall. Three other 1.8 m × 1.8 m plots served as control plots. Soil volumetric water content and soil temperature were monitored twice a week during the field experiment. A digital thermometer and a SM150T moisture sensor (Delta‐T Devices) were inserted carefully at 5 cm depth and measurements were taken between 2 pm and 4 pm in three random locations per plot. The average of these values was reported as the field soil temperature and moisture.

### 2.2 Soil Sampling and Characterization

Soils were sampled in February 2019, at the end of the dry season. Within each plot, five soil samples (0–5 cm depth) were collected, sieved (< 4 mm), and pooled in a composite sample. This resulted in 18 samples: two land uses (forest and cropland), each with three treatments (control, OTC, and shelters), replicated three times. After sampling, the soils were stored in double Ziploc bags and shipped express for analysis.

Soil pH and electrical conductivity were measured in a 1:5 (w:v) soil/water extraction (5 g soil + 25 mL H_2_O) using electrodes (Combined pH Electrodes, Radiometer Analytical, and 4520 Conductivity, Jenway, respectively). Soil organic matter (SOM) was estimated using a loss on ignition procedure (according to Wang et al. 2011). The maximum amount of water that soils could hold after gravity loss was measured to determine the water holding capacity (WHC) of soils (as described in Hicks et al. 2018) (Table S1).

### 2.3 Estimation of Bacterial and Fungal Growth, Respiration and CUE

Bacterial growth was estimated as ^3^H‐leucine incorporation into bacteria (Bååth 1994; Bååth et al. 2001). Briefly, a bacterial suspension was extracted from 0.5 g of soil mixed with 20 mL distilled water. The incorporation of radioactive leucine was done by incubating 1.5 mL of the bacterial suspension with 2 μL radioactively‐labelled leucine ([^3^H]Leu, 185 MBq mL^−1^, 2 TBq mmol^−1^, Perkin Elmer), combined with non‐labelled leucine to a resulting concentration of 280 nM in the sample. After incubation (incubation times adjusted to incubation temperature; see below), growth was terminated by addition of trichlorinated acetic acid. Samples were then washed according to Bååth (1994) and Bååth et al. (2001). The amount of leucine incorporated into extracted bacteria was used as a proxy for bacterial growth. Additionally, ^3^H‐thymidine incorporation was measured simultaneously in samples at 20°C to estimate a conversion factor from leucine to thymidine. From this, the rate of thymidine incorporation was converted to units of bacterial C (Soares and Rousk 2019).

Fungal growth was determined using ^14^C‐acetate incorporation into the fungal‐specific lipid ergosterol (Bååth 2001; Rousk et al. 2009). Briefly, 0.3 g of soil was mixed with 1.95 mL of water, 20 μL of 1‐[^14^C] acetic acid (sodium salt, 37 MBq mL^−1^, 2.10 GBq mmol^−1^, Perkin Elmer) combined with 30 μL of 16 mM non‐labelled sodium acetate; yielding a final concentration of ca. 220 μM in the sample. After incubation, growth was terminated by the addition of formalin, and ergosterol was then extracted, separated, and quantified using high‐performance liquid chromatography and a UV detector (Bååth 2001; Rousk et al. 2009). The ergosterol peak was collected using a fraction collector, and the incorporated acetate was measured using liquid scintillation. The amount of incorporated acetate into extracted ergosterol was used as a proxy for fungal growth, which was expressed in units of fungal C (Soares and Rousk 2019).

For the microbial respiration measurements, 1 g of soil was weighed into 20 mL glass vials, which were purged with pressurized air and sealed with crimp caps. CO_2_ production was measured using a gas chromatograph equipped with a methanizer and a flame ionization detector (YL6500 GC, YL Instruments). Incubation times were also adjusted for the different incubation temperatures (see below).

The resulting CUE was estimated from microbial growth (sum of bacterial growth and fungal growth) divided by the total amount of C used by microorganisms (microbial growth plus respiration).

### 2.4 Estimating Microbial Thermal Trait Distributions Defining the Temperature Relationships for Growth and Respiration

Soils were moisture‐adjusted to 50% WHC and subsequently left in the dark at room temperature (20°C) for 1 week before being processed. This moisture level is optimal for microbial activity and allows isolating temperature effects; while moisture influences the magnitude of microbial growth and respiration, it does not affect their temperature sensitivity (Cruz‐Paredes et al. 2021). Subsamples of the soils were transferred to individual vials that were then exposed to one of ten different temperatures from 0°C to 45°C in 5°C intervals. All samples were processed in technical duplicates. Respiration, bacterial growth and fungal growth were measured for the different soils and temperatures simultaneously. The exposure to different temperatures during the incubation step was always kept short (Table S2). For bacterial growth, an incubation of ~2 h at 20°C–25°C was used, adapting the duration for the other temperatures (i.e., 1 h at 30°C, 4 h at 15°C, etc.) to ensure a similar level of C use in all treatments. Similarly, incubations for fungal growth and respiration were ~4 h and 18 h at 20°C–25°C, respectively, and adapted for other temperatures (i.e., 2 h and 6 h at 30°C, 8 h and 30 h at 15°C, etc.). These incubation times fall within the period during which microbial growth and respiration rates remain stable and are unaffected by altered incubation conditions, apart from the direct effect of temperature (Rousk and Bååth 2011). As such, we determined the “intrinsic temperature relationships” (Davidson and Janssens 2006) based on the Dual‐Kinetics Ratkowsky model (Brangarí and Rousk 2025):

(1a) $$G^{\frac{1}{2}}\left(T\right)=a\left(T-T_{\min}\right)\times \left(1-e^{b\left(T-T_{\max}\right)}\right)$$

(1b) $$R^{\frac{1}{2}}\left(T\right)=a\left(T-T_{\min}\right)\times \left(1+e^{b\left(T-T_{\max}\right)}\right)$$

where *G* is the rate of bacterial growth (*G*_*B*_) or fungal growth (*G*_*F*_), R is the rate of microbial respiration (*R*), *a* and *b* are slope parameters, *T* is the screening temperature (°C), *T*_*min*_ is the minimum temperature (the low x‐axis intercept), and *T*_*max*_ is the maximum temperature (the high x‐axis intercept for growth and its equivalent for respiration).

A simplified version of the model Equations (2a) and (2b) was used for calibrating *T*_*min*_ and the slope *a* on the low temperature range (0°C–25°C),

(2a) $$G^{\frac{1}{2}}\left(T\right)=a\left(T-T_{\min}\right)$$

(2b) $$R^{\frac{1}{2}}\left(T\right)=a\left(T-T_{\min}\right)$$

which were then used as constants in Equations (1a) and (1b) to estimate *T*_*max*_ and *b* using the entire temperature interval. The optimum temperature for growth (*T*_*opt*_) and the equivalent tipping point for respiration (*T*_*tp*_) were estimated equating the derivative of Equations (1a) and (1b) to zero (Brangarí and Rousk 2025).

The *Q*_*10*_ was estimated as an index for the temperature sensitivity as follows:

(3) $$Q_{10}={\left[a\left(\left(T+10\right)-T_{\min}\right)\right]}^{2}/{\left[a\left(T-T_{\min}\right)\right]}^{2}={\left[\left(20-T_{\min}\right)/\left(10-T_{\min}\right)\right]}^{2}$$

where *Q*_*10*_ indicates how the microbial process rates (*G*_*B*_, *G*_*F*_, or *R*) change with a difference of 10°C (here the interval 10°C–20°C was used).

### 2.5 Microbial Community Composition

DNA was extracted from 250 mg of freeze‐dried soil using MoBio Power Soil Kits (MoBio, Carlsbad, CA, USA) following the manufacturer's instructions. DNA was quantified fluorimetrically (Qubit, Invitrogen, Carlsbad, CA, USA). DNA extracts were sent to BGI Tech Solutions (Hong Kong) for HiSeq 2500 PE250 dual index Illumina amplicon sequencing. For bacterial communities, the V3‐V4 region of the 16S was amplified using the primers 341F (5′‐CCTAYGGGRBGCASCAG‐3′) and 806R (5′‐GGACTACNNGGGTATCTAAT‐3′) (Herlemann et al. 2011). For fungal communities, the ITS1‐ITS2 region was amplified using the primers ITS1 (5′‐CTTGGTCATTTAGAGGAAGTAA‐3′) (Gardes and Bruns 1993) and ITS2 (5′‐GCTGCGTTCTTCATCGATGC‐3′) (White et al. 1990). The raw data obtained have been deposited in NCBI with the primary accession number PRJNA1066241. All sequence data were processed using DADA2 and the DADA2 ITS Pipeline Workflow (1.8) (Callahan et al. 2016) to determine the amplicon sequence variants (ASVs) with default settings. The resulting ASVs were used to calculate bacterial and fungal diversity metrics. Sequences were rarefied to the lowest read count using the function *rarefy_even_depth()*. This resulted in a total of 9856 and 3448 ASVs for bacterial and fungal communities, respectively.

### 2.6 Scaling In Situ Estimates and Microbial C Budgets

Bacterial growth, fungal growth and total respiration rates were modeled in situ for 1 year (the entire year before soil sampling) to scale‐up laboratory data and estimate the annual microbial C budget in the field. The intrinsic temperature relationships were used as rate modifiers (Sierra et al. 2015), following approaches commonly applied in decomposition functions in biogeochemical and Terrestrial Ecosystem models (e.g., Bauer et al. 2008; Abramoff et al. 2018). To estimate the rates of growth and respiration over time in the control, OTC and shelter plots, we combined the determined temperature relationships for each field treatment and soil temperature and moisture data recorded in each plot:

(4) $$\text{Bacterial growth}=G_{B}\left(T\right)\times \omega_{B}\left(W\right)=G_{B}\left(T_{\mathrm{opt}}\right)\times \tau_{B}\left(T\right)\times \omega_{B}\left(W\right)$$

(5) $$\text{Fungal growth}=G_{F}\left(T\right)\times \omega_{F}\left(W\right)=G_{F}\left(T_{\mathrm{opt}}\right)\times \tau_{F}\left(T\right)\times \omega_{F}\left(W\right)$$

(6) $$\text{Respiration}=R_{R}\left(T\right)\times \omega_{R}\left(W\right)=R_{R}\left(T_{\mathrm{tp}}\right)\times \tau_{R}\left(T\right)\times \omega_{R}\left(W\right)$$

where $G\left(T_{\mathrm{opt}}\right)$ and $R\left(T_{\mathrm{tp}}\right)$ represents the rates at the tipping‐point or optimal temperature (and moisture), $\tau \left(T\right)$ are the normalized temperature relationships, and $\omega \left(W\right)$ are the normalized moisture relationships, both acting as modulators of temperature and moisture dependence.

Moisture relationships, expressed as a function of volumetric soil water content and based on the DAYCENT model (Kelly et al. 2000; Sierra et al. 2015), were defined as:

(7) $$\omega \left(W\right)={\left(\frac{\frac{W}{100\phi }-c_{2}}{c_{1}-c_{2}}\right)}^{c_{4}\left(\frac{c_{2}-c_{1}}{c_{1}-c_{3}}\right)}{\left(\frac{\frac{W}{100\phi }-c_{3}}{c_{1}-c_{3}}\right)}^{c_{4}}$$

where ϕ is soil porosity, and $c_{1}$, $c_{2}$, $c_{3}$, and $c_{4}$ are fitting parameters determined as 0.6, 1.27, 0.0012, and 2.84, respectively, for fine textured soils (Kelly et al. 2000).

To fill the temperature data gaps and account for diurnal variation in the field, we used a sinusoidal temperature model at the soil surface based on Hillel (1982), calibrated using high‐resolution climate data from the CHELSA database (Karger et al. 2021). The model can thus estimate hourly temperatures based on daily mean temperature values and assumes a symmetric sinusoidal pattern, with the daily temperature range estimated as a linear function of the daily mean, based on empirical calibration using CHELSA data for the study region.

From the rates of microbial growth and respiration over time, we estimated the equivalent rates per unit of soil surface (top 10 cm of soil) and their cumulative annual values. With the estimated in situ soil C fluxes, the microbial CUE of the system (land use and treatment) was estimated, which is a proxy for the soil C budget.

We finally modeled virtual scenarios to assess the direct and indirect effects of the treatments on microbial processes. First, we isolated the direct effect of temperature by using soil temperature data from the treatment plots (OTC or shelter) while keeping moisture data and temperature‐rate relationships from the control plots, thus simulating a scenario where only temperature was altered, without changes in microbial adaptation or moisture. Second, we isolated the direct effect of moisture by using moisture data from the treatment plots while maintaining temperature data and rate relationships from the control plots. Finally, to assess the indirect effects via microbial adaptation, we used temperature and moisture data from the control plots but applied the temperature‐rate relationships from the treatment plots. This three‐step approach allowed us to disentangle the relative contributions of abiotic changes (temperature and moisture) and biotic responses (microbial thermal trait shifts) to the microbial C budget.

### 2.7 Data Analyses

Soil temperature and moisture data measured in the field were separated into dry and wet seasons (as mentioned above). We analyzed the differences between the land use, treatments and their interactions over time (date) in temperature and moisture using a linear mixed effect model with the land use, treatments and date as fixed effects and the sampling plots nested in the blocks as random intercepts to account for the hierarchical study design and the non‐independence of repeated measurements. For pairwise comparisons between land use and treatments, Tukey's HSD test was applied to identify significant differences among interaction means. Two‐way ANOVA with the treatment and land use as factors and block as a random effect was used to identify differences in the microbial growth, respiration, and CUE (at 20°C). We regressed the modeled mean annual soil temperature (Soil MAT; from sinusoidal temperature model, see above) and the maximum temperature in the soil for each plot against *T*_min_, *T*_opt_ or *T*_*tp*_, *T*_*max*_ and *Q*_*10*_ for microbial growth and respiration. The in situ simulations of cumulative microbial growth, respiration, and CUE in the control, shelter and OTC treatment were analyzed separately by land use. For both shelter and OTC treatments, comparisons were made independently against the control and their respective simulations. Statistical significance was assessed using a one‐way ANOVA with block as a random effect.

Alpha diversity was evaluated by calculating the Shannon index, significant differences were calculated with two‐way ANOVA with land use and treatment as factors and block as random effect. Principal coordinate analysis (PCoA) based on Bray‐Curtis dissimilarities was used to visualize beta diversity data. Differences between the treatments and land use were calculated with PERMANOVA analysis using the *adonis2()* function from the vegan package (Oksanen et al. 2015). Further, we used the function *envfit()* from the vegan package (Oksanen et al. 2015) and a mantel test to correlate indices for the bacterial and fungal temperature relationships and other environmental and soil properties with the differences in the bacterial and fungal communities' composition. Finally, we correlated the relative abundance of the different bacterial families and fungal species with soil temperature to identify warm‐adapted microbial taxa. Bacterial abundances were analyzed at the family level because this taxonomic resolution provides a balance between ecological relevance and statistical power, whereas finer taxonomic levels (e.g., genera or ASVs) contain numerous rare taxa that reduce the robustness of correlation analyses. In situ microbial rates and annual fluxes were modeled in MATLAB, while statistical analyses were done in R version 4.0.3 (R Core Team 2020).

## 3 Results

### 3.1 Soil Temperature and Moisture in the Field

Temperature and moisture throughout the year followed typical dynamics of a subtropical climate in northwestern Ethiopia (Tara Gedam site) and exhibited similar patterns in both forest and cropland. During the dry season, soil temperatures ranged between 30°C and 60°C, while soil moisture levels consistently remained below 10% of pore volume saturation (Figure 1). In the wet season, soil temperatures began to decrease with the onset of rainfall, staying between 20°C and 30°C, while soil moisture levels increased significantly, ranging from 20% to 60% of pore volume saturation during July and August (Figure 1). On average, cropland soils were warmer and drier than forest soils (Table S3), with mean dry‐ and wet‐season temperatures of 41.8°C ± 5.2°C and 26.7°C ± 5.1°C (mean ± SD) in cropland compared to 29.8°C ± 3.7°C and 21.9°C ± 3.4°C in forest, and mean soil moisture of 6.1% ± 4.3% and 30.5% ± 12.7% versus 10.5% ± 3.8% and 37.2% ± 14.0%, respectively.

Soil temperature was significantly affected by land use, treatment and their interaction in the dry season (land use: *F* = 831.8, *p* < 0.001, treatment: *F* = 34.5, *p* < 0.001, interaction: *F* = 7.4, *p* = 0.002; Table S3). In the cropland, the OTC treatment increased soil temperature by 3.9°C, from 41.8°C ± 5.2°C to 45.7°C ± 5.8°C, whereas the shelter treatment decreased it by 1.2°C, down to 40.6°C ± 4.2°C (Figure 1A, Table S3). Although the OTC treatment tended to increase soil temperatures, pairwise comparisons were not statistically distinguishable (Figure 1B, Table S3).

Soil moisture was significantly affected by land use, treatment and their interaction in the wet season (land use: *F* = 2.7, *p* = 0.04, treatment: *F* = 6.3, *p* = 0.002, interaction: *F* = 9.3, *p* < 0.001; Table S3). In the cropland, soil moisture did not differ significantly between treatments in either season (Figure 1A, Table S3). In the forest, a treatment effect was observed with the shelter treatment showing a lower level of moisture at 30.4% ± 1.2%, compared to 37.2% ± 1.3% and 35.9% ± 1.3% in the control (*p* < 0.001) and OTC treatment (*p* = 0.003), respectively (Figure 1B, Table S3).

### 3.2 Effects of Field Treatments on Microbial Growth, Respiration, CUE

Direct measurements of bacterial growth, fungal growth, and respiration measured at 20°C, and their resulting CUE showed no significant differences (*p* > 0.05) between treatments or land uses (Figure S1). However, bacterial growth tended (*p* = 0.16) to be higher in the forest compared to the cropland (Figure S1A). Fungal growth was similar in all treatments and land uses (Figure S1B). Respiration tended to be slightly lower in the cropland (*p* = 0.12) and in OTC treatments (*p* = 0.08) (Figure S1C). Finally, CUE was slightly higher in the OTC treatment in the cropland (Figure S1D).

### 3.3 Microbial Thermal Trait Distributions Defining Temperature Relationships

**FIGURE 2:**
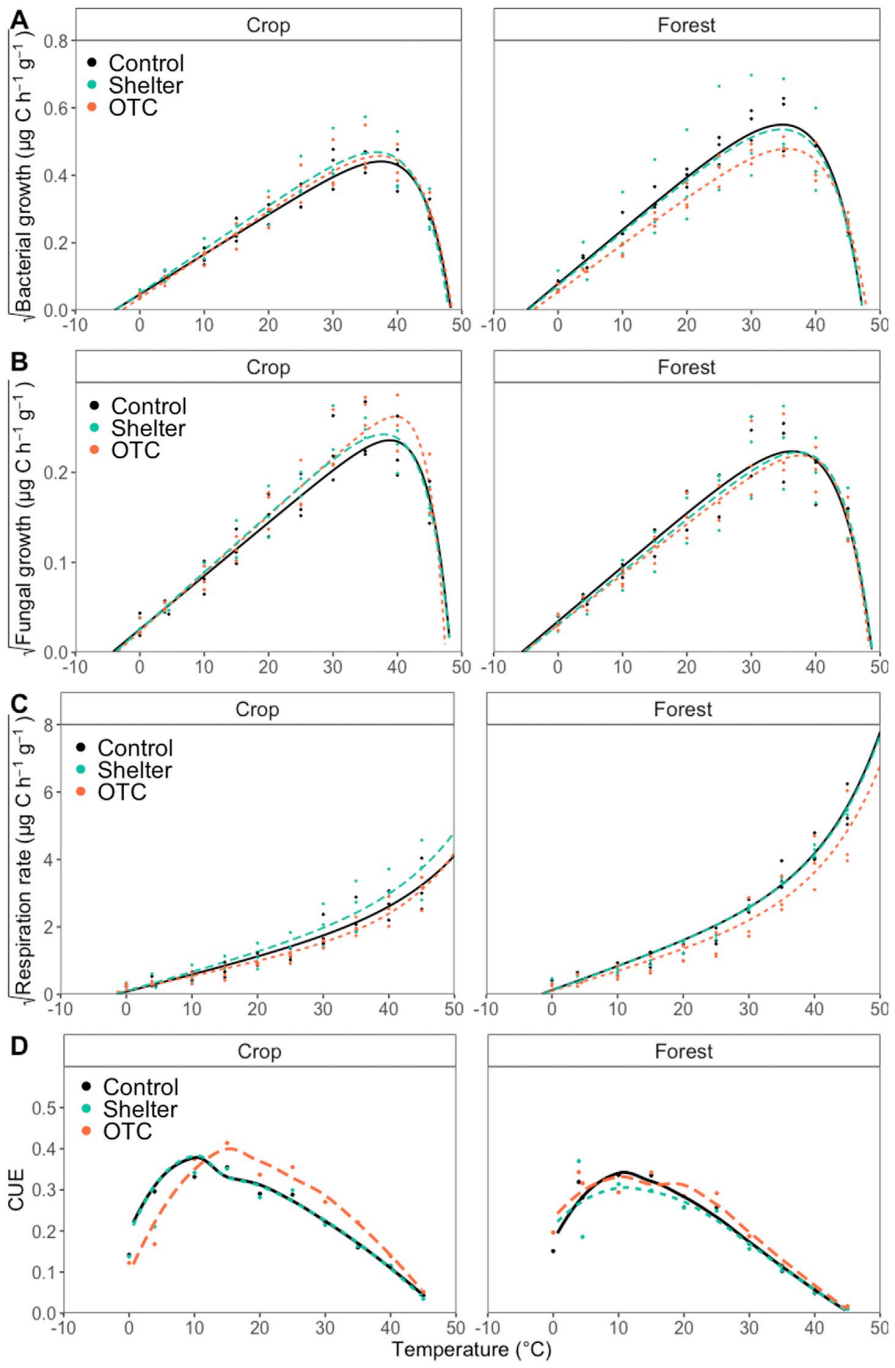
Temperature relationships for square root transformed rates in C units of (A) bacterial growth, (B) fungal growth, (C) respiration, and (D) carbon use efficiency, in the two different land uses (crop and forest). Fitted curves are based on Ratkowsky DK model. Values are the mean of three replicates. Different field treatments are represented with different colors in the two different land uses. These were used to estimate *T*_*min*_, *T*_*opt*_, and *T*_*max*_, which are statistically compared in Figure 3.

Microbial growth rates increased with higher temperatures until an optimum temperature (*T*_opt_) of 33°C to 38°C for bacteria (Figure 2A) and 36°C to 40°C for fungi (Figure 2B). With temperatures over *T*_opt_, rates dropped until a maximum temperature (*T*_max_) of 47°C to 49°C for bacteria and 47°C to 50°C for fungi. All temperature relationships for bacterial and fungal growth were described well by the Ratkowsky DK model, with *R* ^2^ values > 0.93. Respiration rates increased with higher temperatures throughout the entire studied interval, with rates accelerating around *T*_tp_ of 32°C to 40°C (Figure 2C). The temperature relationship curves for respiration were also well captured by the Ratkowsky DK model, with R^2^ values > 0.95. CUE also increased with higher temperatures until approximately 10°C–15°C for cropland and 10°C for forest. After this, CUE decreased across the rest of the temperatures evaluated (Figure 2D).

**FIGURE 3:**
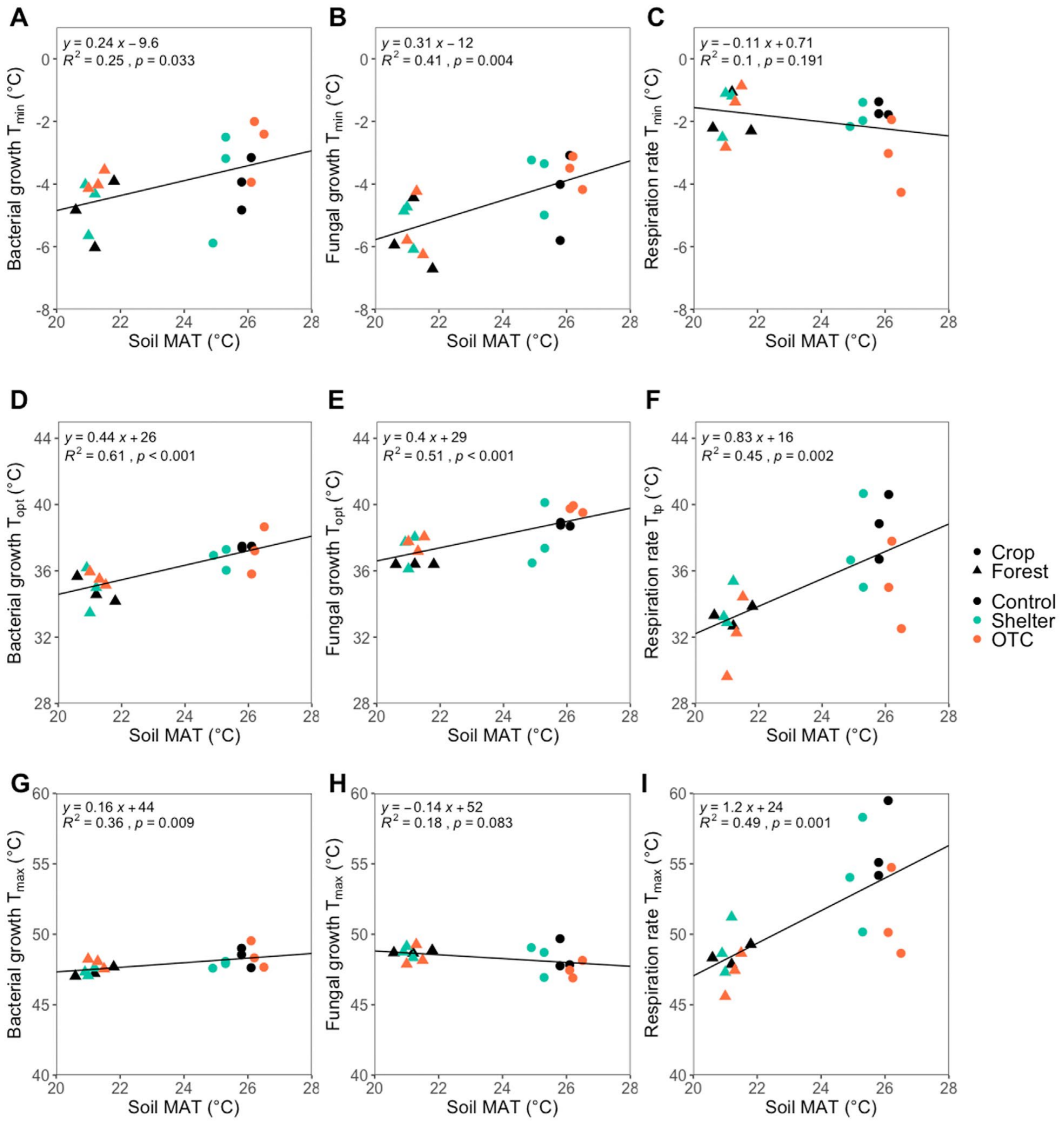
Bacterial growth (A) *T*_*min*_, (D) *T*_*opt*_, (G) *T*_*max*_, fungal growth (B) *T*_*min*_, (E) *T*_*opt*_, (H) *T*_*max*_, and respiration (C) *T*_*min*_, (F) *T*_*tp*_, (I) *T*_*max*_ in the different land uses (crop and forest) and treatments (colors) regressed against soil mean annual temperature in each plot. These parameters were estimated from the temperature relationships for square root transformed rates in Figure 2 using the Ratkowsky DK model.

Bacterial and fungal growth *T*_min_ values increased with higher soil MAT (Figure 3A,B) and higher soil maximum temperature (Figure S2A,B), while no relationships were found for respiration *T*_min_ (Figure 3C, Figure S3C). Bacterial and fungal growth *T*_opt_ values also increased with higher soil temperature (Figure 3D,E; Figure S2D,E), as well as respiration *T*_tp_ (Figure 3F, Figure S2F). Lastly, bacterial growth and respiration *T*_max_ increased with soil MAT (Figure 3G,I) and soil maximum temperature (Figure S2G,I), while no relationships were found for fungal growth *T*_max_ (Figure 3H, Figure S2H). Bacterial and fungal growth *T*_min_ and *T*_opt_ values as well as respiration *T*_tp_ and *T*_max_ were in general higher in the warmer land use, the cropland, while respiration *T*_min_ had the opposite pattern. The temperature sensitivity (*Q*_*10*_) for bacterial growth, fungal growth, and respiration rate did not show any significant correlation with the soil MAT or maximum temperature.

### 3.4 Microbial Diversity, and Community Composition

#### 3.4.1 Bacterial Communities

**FIGURE 4:**
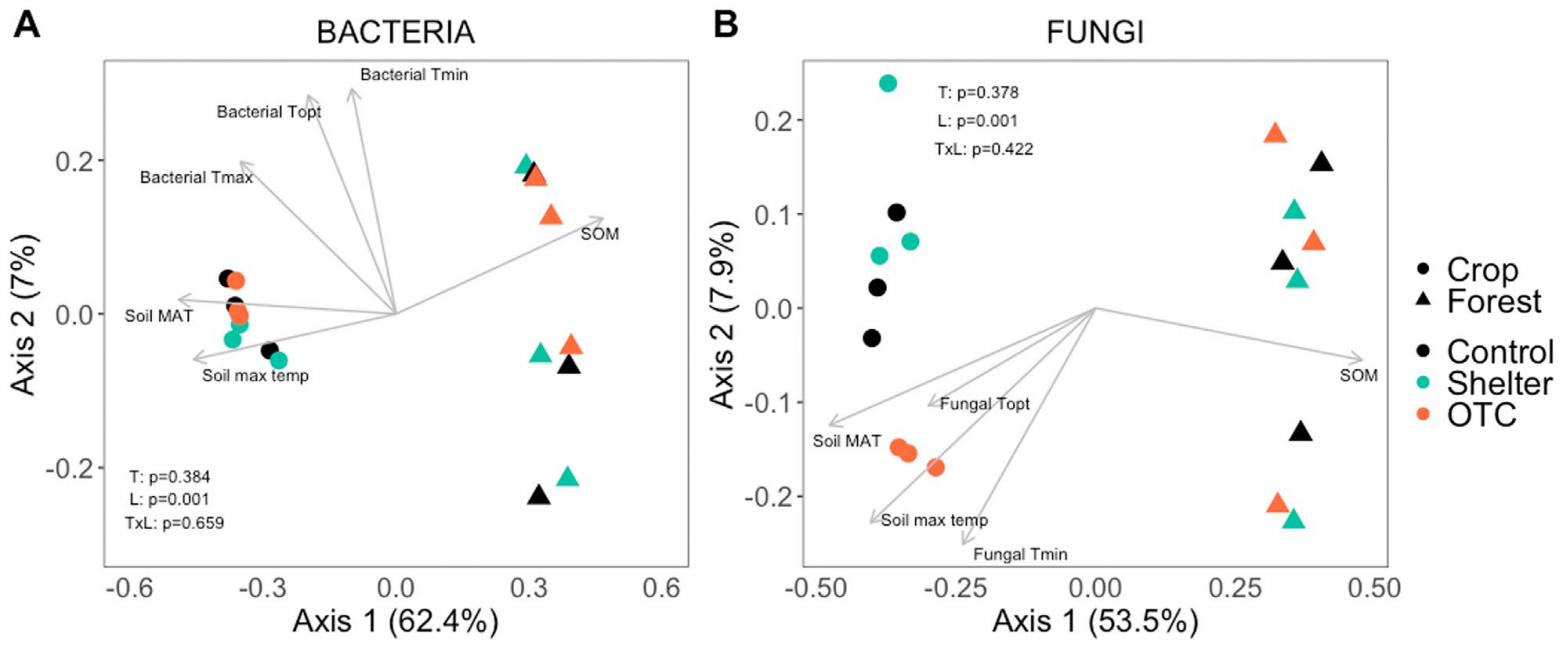
PCoA plots based on Bray‐Curtis dissimilarities of the (A) bacterial and (B) fungal communities in the different land uses, cropland (circles) and forest (triangles) and the different treatments (colors). Arrows represent variables that correlate significantly (*p* < 0.05) with the variance in the communities' composition calculated with the function *envfit* from the vegan package, the length of the arrow represents the *R* ^2^ value.

The bacterial alpha‐diversity, evaluated as Shannon index, did not differ significantly between land uses or treatments (Figure S3A). In contrast, we found that land use significantly influenced the bacterial community composition (*F* = 24.5, *p* = 0.001), while the climate manipulation treatments had no significant effect (Figure 4A). Samples from the different land uses were clustered at different PCoA1 scores, explaining 62.4% of the variation (Figure 4A). We used an unconstrained PCoA to capture overall β‐diversity patterns and applied the *envfit* procedure to test how environmental variables aligned with these gradients. According to the *envfit* function, the bacterial community composition was highly correlated with SOM (*R* ^2^ = 0.93, *p* = 0.001) and soil temperature (max T *R* ^2^ = 0.85, *p* = 0.001; MAT *R* ^2^ = 0.97, *p* = 0.001). Moreover, the three indices for the bacterial temperature relationships were correlated with the bacterial community composition (*T*_min_ *R* ^2^ = 0.38, *p* = 0.02; *T*_opt_ *R* ^2^ = 0.65, *p* = 0.001; *T*_max_ *R* ^2^ = 0.48, *p* = 0.006) and were aligned with the cropland and OTC treatment samples in the PCoA (Figure 4A vectors). The Mantel test also showed significant correlations between the bacterial distance matrix and SOM, soil temperature, and the bacterial temperature relationship indices (Table S4).

Since the variance in the community composition was driven by the differences among land uses (Figure 4A, Axis 1), we separated the data to see if there were differences between treatments within each land use. We found no significant differences between treatments in any of the land uses (Figure S4A,B). However, in the cropland the *envfit* function showed that SOM (*R* ^2^ = 0.87, *p* = 0.004) and the bacterial growth *T*_min_ (*R* ^2^ = 0.84, *p* = 0.003) were correlated with the bacterial community composition (Figure S4A vectors).

Finally, we found that the relative abundance of the bacterial families Caldilineaceae, A4B, BIrii41, Thermoanaerobaculaceae, Chthonomonadaceae, Roseiflexaceae, Kineosporiaceae, Gemmatimonadaceae, Longimicrobiaceae, and Acidobacteriaceae Subgroup 1 had a positive significant correlation with both soil maximum temperature and soil MAT (Table 1).

**TABLE 1:** Pearson correlations of soil mean annual temperature (MAT) and soil maximum temperature (max T) with the relative abundance of bacterial families and fungal species.

	MAT		max T	
	*R* ^*2*^	*p*	*R* ^*2*^	*p*
Bacterial family				
Acidobacteriacea_subgroup 1	0.81	< 0.001	0.72	0.004
Thermoanaerobaculaceae	0.78	0.001	0.75	0.002
Kineosporiaceae	0.83	< 0.001	0.81	< 0.001
Chthonomonadaceae	0.86	< 0.001	0.87	< 0.001
Caldilineaceae	0.89	< 0.001	0.81	< 0.001
A4b	0.91	< 0.001	0.91	< 0.001
Roseiflexaceae	0.88	< 0.001	0.81	< 0.001
Gemmatimonadaceae	0.84	< 0.001	0.80	0.001
Longimicrobiaceae	0.85	< 0.001	0.85	< 0.001
BIrii41	0.86	< 0.001	0.82	< 0.001
Fungal species				
*Setophoma terrestris*	0.84	0.001	0.83	0.002
*Exophiala cancerae*	0.72	0.007	0.64	0.030
*Articulospora proliferata*	0.79	0.003	0.81	0.002
*Fusarium solani*	0.73	0.006	0.68	0.020
*Fusicolla acetilerea*	0.78	0.003	0.73	0.012
*Cryptococcus aspenensis*	0.74	0.006	0.62	0.038

#### 3.4.2 Fungal Communities

The fungal alpha‐diversity did not differ between land use or treatments (Figure S3B). In contrast, the PERMANOVA analysis revealed distinct differences between land uses (*F* = 12.2, *p* = 0.001) but no effect of treatment on the fungal community composition. Samples from the different land uses were clustered at different PCoA1 scores, explaining 53.5% of the variation (Figure 4B). As in the bacterial community, the variance in the fungal community composition was linked to differences in SOM (*R* ^2^ = 0.92, *p* = 0.001) and soil temperature (max T *R* ^2^ = 0.86, *p* = 0.001; MAT *R* ^2^ = 0.97, *p* = 0.001). Similarly, the fungal *T*_min_ and *T*_opt_ were correlated with the fungal community composition (*T*_min_ *R* ^2^ = 0.48, *p* = 0.014; *T*_opt_ *R* ^2^ = 0.40, *p* = 0.015) and aligned with the cropland and OTC treatment samples in the PCoA (Figure 4B, vectors). Additionally, the Mantel test also showed significant correlations between the fungal distance matrix and SOM, pH, soil temperature, and the fungal temperature relationship indices (Table S4).

When analyzing the data within each land use, the PERMANOVA indicated a significant difference among treatments in the cropland (*F* = 1.3, *p* = 0.03). The warming treatment samples were clustered at different PCoA1 and PCoA2 scores, explaining 24.9% and 21.5% of the variation, respectively (Figure S4C). This separation was linked to differences in soil temperature (max T *R* ^2^ = 0.84, *p* = 0.003; MAT *R* ^2^ = 0.66, *p* = 0.036) and fungal *T*_opt_ (*R* ^2^ = 0.78, *p* = 0.011) (Figure S4C vectors). Additionally, SOM correlated negatively to the OTC treatments (*R* ^2^ = 0.68, *p* = 0.025) (Figure S4C vector). Within the forest site, we did not find any significant difference between treatments (Figure S4D). However, two indices for the fungal temperature relationship (*T*_*min*_ *R* ^2^ = 0.61, *p* = 0.048; *T*_max_ *R* ^2^ = 0.67, *p* = 0.038) correlated with the variation in the fungal community composition (Figure S4D, vectors).

Finally, we found that the relative abundance of the fungal species *Articulospora proliferate*, and *Fusicolla acetilerea*, as well as the pathogenic fungal species *Setophoma terrestris*, *Exophiala cancerae*, *Cryptococcus aspenensis*, and *Fusarium solani* had a positive significant correlation with both soil maximum temperature and soil MAT (Table 1).

### 3.5 Simulations of In Situ Soil C Budgets

**FIGURE 5:**
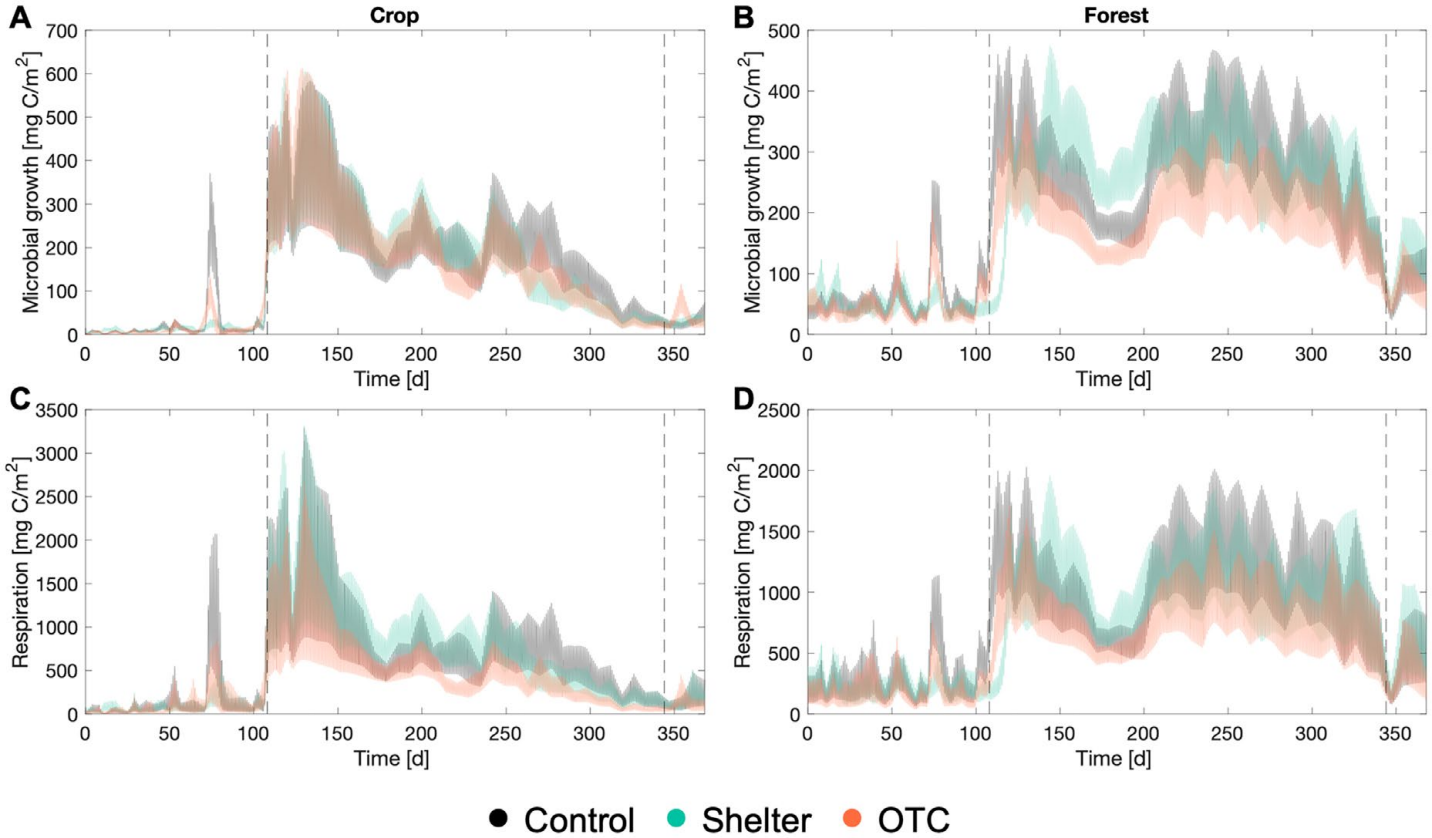
Simulated in situ rates of microbial growth (A, B) and soil respiration (C, D) during a year in the two land uses: Crop (A, C) and forest (B, D) sites. Different colors represent different treatments.

**FIGURE 6:**
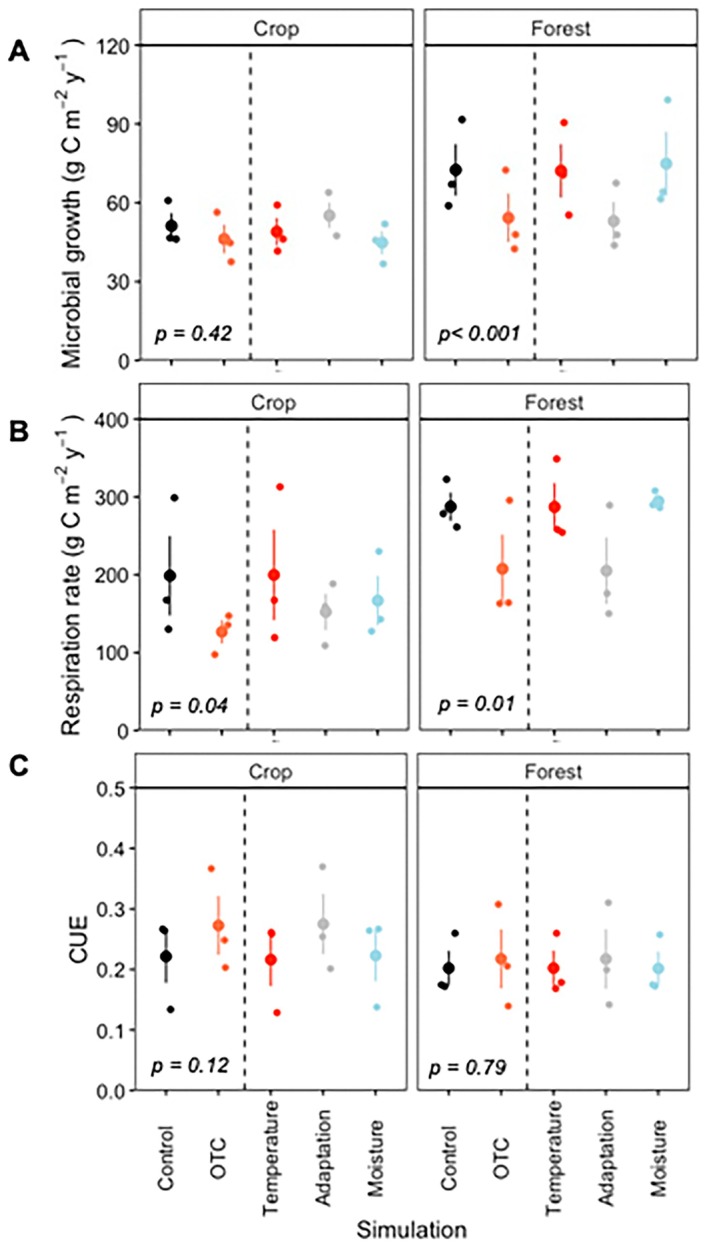
Simulations of cumulative (A) microbial growth, (B) soil respiration, and (C) carbon use efficiency in the different land‐uses (crop and forest). Values are mean values (*n* = 3) and errors bars represent standard errors. Dots to the right of the dashed lines show the separated effect of temperature, microbial adaptation and moisture in the OTC treatment. The *p* values indicate the significance of the differences between simulations.

The modeled in situ growth and respiration rates were highly sensitive to seasonal environmental variation, showing pronounced differences between dry and wet periods and between the two land uses (Figure 5). Cumulative annual field fluxes indicated that overall microbial growth and respiration were higher in forest soils than in croplands (Figure 6, Figure S5). Treatment effects were generally small and often masked by substantial variability among replicates. However, plots subjected to the OTC treatment consistently exhibited lower microbial process rates than controls (Figure 6), whereas the shelter treatment showed no discernible effect in either land use type (Figure S5). In the cropland, modeled cumulative microbial growth showed only minor differences between control and OTC treatments (Figure 6A). In contrast, cumulative respiration decreased significantly (*F* = 4.1, *p* = 0.04), declining from 199 g C m^−2^y^−1^ in the control to 127 g C m^−2^y^−1^ under OTC conditions (Figure 6B). Since respiration declined more than growth, CUE exhibited an increasing trend (*F* = 2.6, *p* = 0.12), rising from 0.22 in the control to 0.27 in the OTC treatment (Figure 6C). In the forest, cumulative microbial growth decreased significantly in the OTC treatment (*F* = 40.2, *p* < 0.001), from 72.5 g C m^−2^y^−1^ in the control to 54.3 g C m^−2^y^−1^. Similarly, cumulative respiration declined significantly (*F* = 6.5, *p* = 0.01), from 287 g C m^−2^y^−1^ to 208 g C m^−2^y^−1^. Since both growth and respiration decreased proportionally in the forest soils, CUE remained unchanged between treatments (Figure 6C). Finally, due to the minor changes in microbial rates induced by the shelter, this treatment had no measurable effect on CUE in either of the land use types (Figure S5).

A more detailed analysis of the direct and indirect drivers of microbial responses (bars to the right of the dotted line in Figure 6, Figure S5) revealed that the observed reductions in microbial rates were primarily attributable to differences in the thermal traits of microbial communities (grey bars, simulation: Adaptation). In contrast, the minor OTC‐induced changes in soil temperature and moisture (Figure 1, Table S3) resulted in negligible direct effects on microbial rates (Figures S1 and S6). This was further supported by the modeled rates based solely on altered temperature (red bars) and moisture (blue bars), which showed no substantial deviation from control values (black bars) (Figure 6). Notably, even the modest shifts in microbial thermal traits (Figure 2), when considered independently of environmental changes, were sufficient to drive the observed declines in microbial growth and respiration.

## 4 Discussion

Our results show that the effects of warming and shelter treatments on soil temperature were strongly dependent on land use, with more pronounced responses in the cropland and limited effects in the forest. The OTC treatments slightly reduced annual levels of respiration rates after 1.5 years in both croplands and forests. Additionally, bacterial growth rates were lower in the cropland site, which was characterized by warmer soil temperatures, consistent with suppressed microbial activity under warmer temperatures. However, site differences in temperatures also coincided with differences in SOM content and moisture levels, raising the possibility that differences in microbial process rates could be the outcome of both direct and indirect effects of temperatures, including differences in substrate availability and water dynamics (Conant et al. 2011; Hartley et al. 2007; Kirschbaum 2004; Melillo et al. 2002). To address this challenge, we combined determinations of microbial thermal trait distributions with field measurements of temperature and moisture through modeling, allowing us to assign responses in microbial process rates to their causes and infer their implications for soil C budgets.

Although absolute microbial growth and respiration rates did not differ significantly across treatments (Figure S1), the temperature responses still varied, indicating that shifts in thermal trait distributions can occur independently of changes in activity levels. We found that sites with warmer soil temperatures were associated with warm‐shifted thermal trait distributions, as indicated by higher *T*_min_ and *T*_opt_ values for both bacterial and fungal growth, as well as higher *T*_tp_ and *T*_max_ values for respiration (Figure 3). Previous studies have reported that environmental temperatures force thermal trait distributions to change, where *T*_min_ values increase by approximately 0.2°C to 0.3°C for every 1°C rise in MAT (Rinnan et al. 2009; Rousk et al. 2012; van Gestel et al. 2013; Bååth 2018; Alster et al. 2023). Across our full dataset, our results are consistent with this, as we find that thermal traits shifted by 0.24°C increase in bacterial growth *T*_min_ per 1°C difference in soil MAT (*R* ^2^ = 0.25, *p* = 0.03), and a 0.31°C increase for fungal growth *T*_min_ (*R* ^*2*^ = 0.41, *p* = 0.004). These thermal estimates also match patterns for the environmental forcing of thermal trait distributions observed in studies along altitudinal temperature gradients in tropical regions (Nottingham et al. 2019), warming field experiment in temperate ecosystems (Rousk et al. 2012), latitudinal gradients across Europe (Cruz‐Paredes et al. 2023), and geothermal gradients across New Zealand (Alster et al. 2023). Our results therefore extend the generalizability of the environmental forcing of thermal trait distributions, to also apply to intensively managed and pristine subtropical ecosystems. However, the effective warming of the OTC‐treatments within each site were variable, resulting in tendencies for subtle differences in temperature relationships (Figure 2), but not manifesting as significant increases in the indices used to track changes in thermal trait distributions (Figure 3).

It is possible that the OTC‐treatment effects on environmental temperatures were subtle and only manifested during the warm (and also very dry) season (Figure 1, Table S3), rendering soil MAT an ambiguous indicator for environmental forcing. Indeed, it was recently shown that fluctuating temperatures, rather than sustained warming, resulted in reduced microbial respiration responses and reduced temperature sensitivities in subtropical forests (Zhang et al. 2023). It should also be noted that administering warming with OTCs can alter microclimatic factors such as airflow and precipitation interception (Hollister et al. 2023), introducing alternative drivers for change of microbial rates and communities in addition to temperature. The effect of the field experiments treatments on environmental conditions also varied between land‐use types, raising challenges for discerning generalizable warming effects. In the forest plots, OTCs resulted in a warming of +0.9°C, with the extensive shading of the canopy probably limiting effect sizes. In contrast, in the cropland plots, characterized by open exposure, OTCs resulted in a pronounced warming effect of +3.9°C. Nonetheless, in our study OTCs did not generate measurable changes in soil moisture (Table S3), suggesting that their main influence was on temperature rather than hydrology under these specific site conditions.

The adaptation of microbial thermal trait distributions to environmental temperatures can be attributed to several co‐occurring mechanisms: (i) physiological acclimation, where individual microbes adjust their cellular physiology to cope with higher temperatures; (ii) evolutionary adaptation, where traits shift within populations of the same species; and (iii) species sorting, where species that perform better under warmer conditions outcompete others (Bárcenas‐Moreno et al. 2009; Bradford 2013). The rate of change for each of these possibilities should scale with metabolic rates, enabling changes in physiology, generational turnover, or succession. That the warmest environmental temperatures consistently occur when soils are also very dry in the studied ecosystems means that overall microbial process rates would be low (Zhang et al. 2013), but likely exceed zero. With low rates of metabolism, community trait adaptation could still occur, although it would be slow. Specifically, modeled microbial growth rates during the dry season averaged about 11% and 25% of wet‐season values in the crop and forest, respectively (Figure 5), resulting in 9‐ and 4‐fold estimated slower changes in thermal trait distributions. As such, thermal trait distribution shifts still occurred under seasonal warming, but changes were insufficient to produce significant differences across treatments (Figure 3). Moreover, we found that thermal trait distributions did not align with the full span of environmental temperatures observed in the field, nor with the maximum annual soil temperatures. Unlike previous reports for ecosystems with high soil moisture during seasonal temperature peaks (Donhauser et al. 2020; Tájmel et al. 2024), where *T*_opt_ closely approach the maximum recorded soil temperatures, our findings show a different pattern. In the studied sites, microbial thermal trait distributions failed to cover the entire environmental temperature range, and *T*_opt_ remained substantially lower than the peak soil temperatures measured in situ (Figure S2). This contrast may reflect a fundamental difference in systems where temperature and moisture availability are temporally offset.

Differences in the microbial thermal trait distribution indices *T*_min_, *T*_opt_, and *T*_max_ coincided with differences in microbial community composition (Figure 4, Figure S4). These results suggest that species sorting contributed to the differences in thermal trait distributions, where taxa performing better under warmer conditions increased in relative abundance under elevated temperatures (Donhauser et al. 2020; Rijkers et al. 2022). Similar patterns have recently been reported in subtropical forest, where long‐term warming shifted the microbial community toward more K‐strategists adjusting microbial respiration and growth to pre‐warming levels within a few seasons (Liu et al. 2025). Bacterial and fungal community composition also varied markedly between the land uses. Cropland soils experienced 7°C (wet season) to 10°C (dry season) warmer temperatures than forest soils. Land use changes are known to alter key soil properties such as pH, nutrient availability, and structure, all of which can influence microbial community composition and function (Delelegn et al. 2018; Lauber et al. 2008). Additionally, differences in vegetation, such as plant diversity, plant cover, and plant functional traits, can strongly shape soil microbial communities (Delgado‐Baquerizo et al. 2018). In our study, besides the differences generated by land use, the compositional shifts in bacterial and fungal communities were primarily associated with variations in soil temperature and SOM, highlighting their role as key drivers of microbial community structure (Figure 4). This is consistent with previous works showing that soil temperature strongly regulates microbial metabolic rates, enzyme activity, and growth strategies, favoring taxa with higher thermal optima under warming (Bradford 2013; Eng et al. 2023; Wang et al. 2021). SOM, in turn, can act as both an energy source and as a structural habitat, influencing microbial diversity and CUE (Domeignoz‐Horta et al. 2020; Lehmann and Kleber 2015). Moreover, correlations between thermal trait indices and community composition indicate that temperature‐related traits are closely linked to community assembly processes. This suggests that environments experiencing higher temperatures, such as cropland and OTC treatments, appear to select for taxa with higher thermal optima, reflecting an adaptive response to warming and land‐use intensification (Figure 4). In croplands, the fungal communities in the OTC treatment differed significantly from those in control and shelter treatments (Figure S4C). These distinct responses likely resulted from the combined effects of altered SOM and temperature regimes, reinforcing the idea that thermal adaptation interacts with resource availability to shape microbial communities. This aligns with evidence from large‐scale subtropical transects showing that microbial temperature sensitivity varies strongly across climate zones, with warming reducing cold‐preferring taxa and altering SOC pool stability (Li et al. 2024).

We also observed taxon‐specific responses to warming. Several bacterial families with known thermophilic or thermotolerant traits, such as Caldilineaceae, Thermoanaerobaculaceae, Chthonomonadaceae, and Roseiflexaceae, became more abundant at higher temperatures (Dedysh and Yilmaz 2018; Hanada et al. 2002; Lee et al. 2011). Similarly, Acidobacteria and Gemmatimonadaceae, recognized as warm‐adapted groups (Oliverio et al. 2017), increased in abundance with rising temperatures. Their increase suggests that warming may shift community composition towards taxa capable of sustaining activity under thermal stress, potentially altering decomposition dynamics and nutrient turnover (Li et al. 2024). For fungi, higher soil temperatures favored pathogenic taxa such as *Fusicolla acetilerea* and *Fusarium solani*, consistent with previous studies reporting that warming facilitates thermotolerant and pathogenic fungal groups (Cuartero et al. 2024; Geml et al. 2015; Nottingham et al. 2022; Semenova et al. 2015). According to Singh et al. (2023), climate warming may enhance pathogen abundance by altering their behavior, expanding their geographic range, and facilitating the emergence of new pathogenic strains. Such shifts may influence plant–soil interactions by increasing disease pressure or modifying saprotrophic activity, ultimately affecting ecosystem productivity and C cycling (Pec et al. 2021).

Our study also showed that microbial temperature trait distributions are matched to local temperature regimes, indicating that static temperature rate modifiers cannot accurately represent differences across natural ecosystems and climates. To assess these implications, we used the measured temperature trait distributions along with in situ moisture and temperature data to evaluate in situ microbial rates and microbially driven C cycling dynamics, upscaling laboratory data to the field (Figure 5). Direct validation of the modeled in situ respiration rates with field CO_2_ flux measurements would have strengthened our analysis. However, such comparisons are not completely appropriate because chamber measurements integrate microbial and autotrophic respiration, and CO_2_ produced from deeper soil layers, whereas our model estimates only microbial heterotrophic respiration in the top 10 cm. We therefore highlight the need for such validation in future work. In our modeling approach, we observed contrasting responses in soil C budgets between cropland and forest land uses. In the cropland, the OTC treatment had no apparent effect on annual cumulative microbial growth but moderately reduced annual respiration from 199 g C m^−2^y^−1^ to 127 g C m^−2^y^−1^, leading to a slight increase in CUE from 0.22 in the control to 0.27 (Figure 6; black vs. orange bars). In the forest, both microbial growth and respiration declined from 72.5 g C m^−2^y^−1^ to 54.3 g C m^−2^y^−1^ and from 287 g C m^−2^y^−1^ to 208 g C m^−2^y^−1^ in the OTC treatment, respectively (Figure 6). Consequently, the microbial CUE remained similar in both the OTC treatment and the control, suggesting no apparent implications for SOC stocks. The contrasting results in cropland and forest can be attributed to variations in soil conditions, vegetation type, and resource availability (Delgado‐Baquerizo et al. 2018; Lauber et al. 2008). Land use also influences the susceptibility of soils to drying (Olorunfemi and Fasinmirin 2017), which in turn also affected how environmental conditions changed in response to the different experimental treatments. Soil temperature and moisture differed across land uses, with a cooler and moister environment in the forest (Figure 1). In contrast, the cropland was exposed to drier conditions and more severe moisture deficit which can affect microbial physiology and activity (Manzoni et al. 2012). Consequently, land‐use‐generated differences in soil moisture may create a higher potential for microbial growth after rewetting in the cropland than forest.

Our estimates of in situ C budgets revealed that the direct effect of treatment‐induced changes in temperature and moisture alone had no effect on cumulative microbial growth and respiration (Figure 6, red and blue bars compared to the black bar). This finding contrasts with a previous study using a similar modeling approach (Brangarí et al. 2025), where a consistent +5°C warming throughout the year, without significant moisture limitation, directly enhanced microbial growth and respiration rates by approximately 50%. In that study, the gradual adaptation of thermal traits over 9 years eventually reduced heat‐induced emissions back to pre‐heating levels and growth rates to about halfway, explaining the observed changes in SOC stocks. The absence of direct temperature effects on annual microbial rates (Figure 6, red bars) can likely be attributed to the characteristics of the treatments, which produced seasonally inconsistent temperature effects, and to the extreme climatic conditions typical of subtropical ecosystems, where maximum temperatures coincide with low soil moisture (Figure 1). Despite no direct effect of OTC‐induced differences in temperature on annual rates, the subtle changes did lead to an overall reduction of both microbial growth and respiration rates (Figure 6, orange bars). This highlights the critical role of microbial thermal trait adaptation in regulating C cycling and soil functioning. It also reveals that strong moisture constraints of rates of metabolism required to support trait distribution changes will modulate the influence of these thermal traits on microbial responses, demonstrating the complexity of predicting ecosystem responses under climate change. Our findings thus suggest that accurate future projections will require both characterization of microbial thermal trait distributions along with both temperature and rainfall patterns providing the environmental forcing of microbial processes.

## 5 Conclusion

We found that microbial communities in warmer sites exhibit warm‐shifted thermal trait distributions, characterized by higher thermal indices (*T*_min_, *T*_opt_, and *T*_*max*_) for microbial growth and respiration. These differences in microbial thermal trait distributions were strongly associated with changes in community composition, particularly in warmer environments. The microbial compositional changes reflected a predominance of thermotolerant bacterial and fungal taxa, including known heat‐adapted and pathogenic groups, suggesting that rising temperatures selectively favor organisms with traits suited to warmer conditions. However, thermal indices (*T*_opt_) did not align with peak seasonal temperatures, as severe moisture limitations coincided with the warmest periods, likely preventing consistent trait shifts across treatments. Finally, the warm adaptation of microbial thermal trait distributions contributed to mitigating ecosystem C losses in the cropland by reducing respiration rates by 36% in the OTC treatment. This functional adjustment promoted C sequestration under warming scenarios in a subtropical ecosystem, highlighting the potential for microbial thermal trait adaptation to buffer climate‐driven impacts on soil C dynamics.

## Author Contributions

**Carla Cruz‐Paredes:** conceptualization, funding acquisition, data curation, formal analysis, investigation, methodology, visualization, writing – original draft, writing – review and editing. **Albert C. Brangarí:** writing – review and editing, formal analysis, data curation, writing – original draft, investigation, visualization. **Dániel Tájmel:** writing – review and editing, investigation, methodology. **Lettice Hicks:** conceptualization, investigation, methodology, writing – review and editing. **Ainara Leizeaga:** conceptualization, investigation, methodology, writing – review and editing. **Menale Wondie:** conceptualization, funding acquisition, investigation, methodology, project administration, resources, supervision, writing – review and editing. **Hans Sandén:** conceptualization, funding acquisition, investigation, project administration, supervision, writing – review and editing. **Johannes Rousk:** conceptualization, funding acquisition, investigation, methodology, project administration, resources, supervision, writing – review and editing.

## Funding

This work was supported by Vetenskapsrådet (2016‐06327); Danmarks Frie Forskningsfond (9036‐00004B); Knut och Alice Wallenbergs Stiftelse (KAW 2022.0175 and KAW 2023.0384).

## Conflicts of Interest

The authors declare no conflicts of interest.

## Supporting information


**Table S1:** Soil characteristics in the different land‐uses and treatments. Values represent means (*n* = 3) ± standard errors.
**Table S2:** Subsamples of the soils were transferred to vials that were exposed to ten different temperatures from 0°C to 45°C in 5°C intervals in water baths. The exposure to different temperatures during the incubation step was adapted for the different temperatures as follows:
**Table S3:** Mean values ± standard deviation of soil temperature (°C) and moisture (%) in dry and wet seasons across land‐uses and treatments. Different letters denote significant differences among interaction means (land‐use × treatment) based on Tukey's test (*p* < 0.05).
**Table S4:** Correlations between environmental variables and distance matrices for bacterial and fungal communities using mantel test with Pearson correlation. Significant values are in bold.
**Figure S1:** Effects of field experimental treatments (colors) on (A) bacterial growth, (B) fungal growth, (C) respiration rate and (D) carbon use efficiency, evaluated at 20°C in the different land‐uses (crop and forest). Values are mean values (*n* = 3) and errors bars represent standard errors.
**Figure S2:** Bacterial growth (A) *T*_*min*_, (D) *T*_*opt*_, (G) *T*_*max*_, fungal growth (B) *T*_*min*_, (E) *T*_*opt*_, (H) *T*_*max*_ and respiration rate (C) *T*_*min*_, (F) *T*_*tp*_, (I) *T*_*max*_, in the different land uses (crop and forest) and treatments (colors) regressed against soil maximum temperature in each plot.
**Figure S3:** Soil microbial communities' alpha diversity for (A) bacterial and (B) fungal communities reported as Shannon diversity index in the different land‐uses (crop and forest) and treatments (colors). Values are mean values (*n* = 3) and errors bars represent standard errors.
**Figure S4:** PCoA plots based on Bray‐Curtis dissimilarities of the bacterial (A, B) and fungal (C, D) communities separated by land‐uses, crop (A, C), and forest (B, D) to reveal possible separations between treatments (colors). Arrows represent variables that correlate significantly (*p* < 0.05) with the variance in the communities' composition calculated with the function *envfit* from the vegan package, the length of the arrow represents the *R* ^2^ value.
**Figure S5:** Simulations of cumulative (A) microbial growth, (B) respiration, and (C) carbon use efficiency in the different land‐uses (crop and forest). Values are mean values (*n* = 3) and errors bars represent standard errors. Dots to the right of the dashed lines show the separated effect of temperature, microbial adaptation, and moisture in the shelter treatment. The *p* values indicate the significance of the differences between simulations.

## Data Availability

All data and code supporting the findings of this study are openly available on Figshare at: https://doi.org/10.6084/m9.figshare.31746121. Sequence data have been deposited in the National Center for Biotechnology Information (NCBI) under BioProject accession number PRJNA1066241: https://www.ncbi.nlm.nih.gov/bioproject/PRJNA1066241.
