# Supplementary material for: Soil Microbial Communities Adjust Thermal Traits and Carbon Allocation in Response to Climate Manipulations in Subtropical Forest and Cropland
Source: Glob Chang Biol. 2026 Apr 4;32(4):e70836. doi: 10.1111/gcb.70836 (PMC13049464; doi:10.1111/gcb.70836)
Supplement: Supplementary file 1 — Table S1: Soil characteristics in the different land‐uses and treatments. Values represent means (n = 3) ± standard errors. Table S2: Subsamples of the soils were transferred to vials that were exposed to ten different temperatures from 0°C to 45°C in 5°C intervals in water baths. The exposure to different temperatures during the incubation step was adapted for the different temperatures as follows: Table S3: Mean values ± standard deviation of soil temperature (°C) and moisture (%) in dry and wet seasons across land‐uses and treatments. Different letters denote significant differences among interaction means (land‐use × treatment) based on Tukey's test (p < 0.05). Table S4: Correlations between environmental variables and distance matrices for bacterial and fungal communities using mantel test with Pearson correlation. Significant values are in bold. Figure S1: Effects of field experimental treatments (colors) on (A) bacterial growth, (B) fungal growth, (C) respiration rate and (D) carbon use efficiency, evaluated at 20°C in the different land‐uses (crop and forest). Values are mean values (n = 3) and errors bars represent standard errors. Figure S2: Bacterial growth (A) T min , (D) T opt , (G) T max , fungal growth (B) T min , (E) T opt , (H) T max and respiration rate (C) T min , (F) T tp , (I) T max , in the different land uses (crop and forest) and treatments (colors) regressed against soil maximum temperature in each plot. Figure S3: Soil microbial communities' alpha diversity for (A) bacterial and (B) fungal communities reported as Shannon diversity index in the different land‐uses (crop and forest) and treatments (colors). Values are mean values (n = 3) and errors bars represent standard errors. Figure S4: PCoA plots based on Bray‐Curtis dissimilarities of the bacterial (A, B) and fungal (C, D) communities separated by land‐uses, crop (A, C), and forest (B, D) to reveal possible separations between treatments (colors). Arrows represent variables that correla [file GCB-32-e70836-s001.docx]

**Supplementary material for**

**Soil microbial communities adjust thermal traits and carbon allocation in response to climate manipulations in subtropical forest and cropland**

Carla Cruz-Paredes*, Albert C. Brangarí, Dániel Tájmel, Lettice Hicks, Ainara Leizeaga, Menale Wondie, Hans Sanden, Johannes Rousk

*corresponding author

**Content:**

**Figures S1 – S5**

**Tables S1 – S4**

**Table S1.** Soil characteristics in the different land-uses and treatments. Values represent means (n=3) ± standard errors.

| **Treatment** | **pH** | **EC** | **% SOM** | **100% WHC** |
| --- | --- | --- | --- | --- |
| Crop |  |  |  |  |
| Control | 6.8 ± 0.03 | 46 ± 1.2 | 8.8 ± 0.15 | 71 ± 1.1 |
| Shelter | 6.4 ± 0.03 | 52 ± 4.2 | 9.3 ± 0.39 | 72 ± 3.8 |
| OTC | 6.7 ± 0.21 | 46 ± 4.7 | 8.6 ± 0.13 | 70 ± 1.6 |
| Forest |  |  |  |  |
| Control | 6.7 ± 0.03 | 66 ± 3.1 | 18.9 ± 0.80 | 85 ± 1.0 |
| Shelter | 6.7 ± 0.04 | 71 ± 3.7 | 19.5 ± 1.53 | 84 ± 3.3 |
| OTC | 6.7 ± 0.06 | 61 ± 6.3 | 18.5 ± 0.67 | 80 ± 1.7 |

**Tables S2.** Subsamples of the soils were transferred to vials that were exposed to ten different temperatures from 0°C to 45°C in 5°C intervals in water baths. The exposure to different temperatures during the incubation step was adapted for the different temperatures as follows:

| Incubation temperature | Bacterial growth | Fungal growth | Respiration rate |
| --- | --- | --- | --- |
| 0°C | 32 h | 64 h | 149 h |
| 5°C | 16 h | 32 h | 80 h |
| 10°C | 8 h | 16 h | 48 h |
| 15°C | 4 h | 8 h | 30 h |
| 20°C | 2 h | 4 h | 18 h |
| 25°C | 2 h | 4 h | 18 h |
| 30°C | 1 h | 2 h | 6 h |
| 35°C | 1 h | 2 h | 3 h |
| 40°C | 1 h | 2 h | 3 h |
| 45°C | 1 h | 2 h | 3 h |

These times ensure a similar level of C use in all treatments, and within these time periods no change in growth rates due to altered growth conditions occurs, except for the direct effect of temperature on rates.

| **CROP Temperature** | **Dry season** | | | **Wet season** | | |
| --- | --- | --- | --- | --- | --- | --- |
| Control | 41.8 | ± 5.2 | B | 26.7 | ± 5.1 | A |
| Shelter | 40.6 | ± 4.2 | B | 25.6 | ± 4.6 | A |
| OTC | 45.7 | ± 5.8 | A | 25.8 | ± 5.3 | A |
| **FOREST Temperature** | | | | | | |
| Control | 29.8 | ± 3.7 | C | 21.9 | ± 3.4 | B |
| Shelter | 28.9 | ± 2.5 | C | 22.0 | ± 3.3 | B |
| OTC | 30.7 | ± 4.0 | C | 21.7 | ± 3.4 | B |
|  | | | | | | |
| **CROP Moisture** | | | | | | |
| Control | 6.1 | ± 4.3 | B | 30.5 | ± 12.7 | B |
| Shelter | 5.4 | ± 1.8 | B | 27.7 | ± 13.5 | B |
| OTC | 5.3 | ± 2.8 | B | 26.6 | ± 12.3 | B |
| **FOREST Moisture** | | | | | | |
| Control | 10.5 | ± 3.8 | A | 37.2 | ± 14.0 | A |
| Shelter | 9.8 | ± 2.8 | A | 30.4 | ± 13.0 | B |
| OTC | 10.0 | ± 4.3 | A | 35.9 | ± 13.0 | A |
|  |  |  |  |  |  |  |

**Table S3.** Mean values ± standard deviation of soil temperature (°C) and moisture (%) in dry and wet seasons across land-uses and treatments. Different letters denote significant differences among interaction means (land-use x treatment) based on Tukey’s test (p<0.05).

##

T: p= 0.54

L: p= 0.16

TxL: p= 0.53

T: p= 0.90

L: p= 0.96

TxL: p= 0.13

T: p= 0.08

L: p= 0.12

TxL: p= 0.59

T: p= 0.38

L: p= 0.23

TxL: p= 0.85


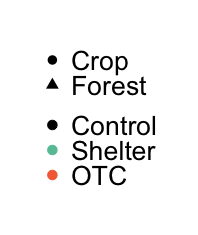


**Figure S1.** Effects of field experimental treatments (colors) on (A) bacterial growth, (B) fungal growth, (C) respiration rate and (D) carbon use efficiency, evaluated at 20°C in the different land-uses (crop and forest). Values are mean values (n= 3) and errors bars represent standard errors.

**
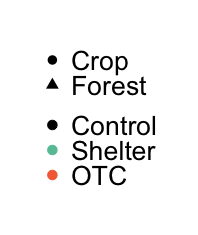
**

**
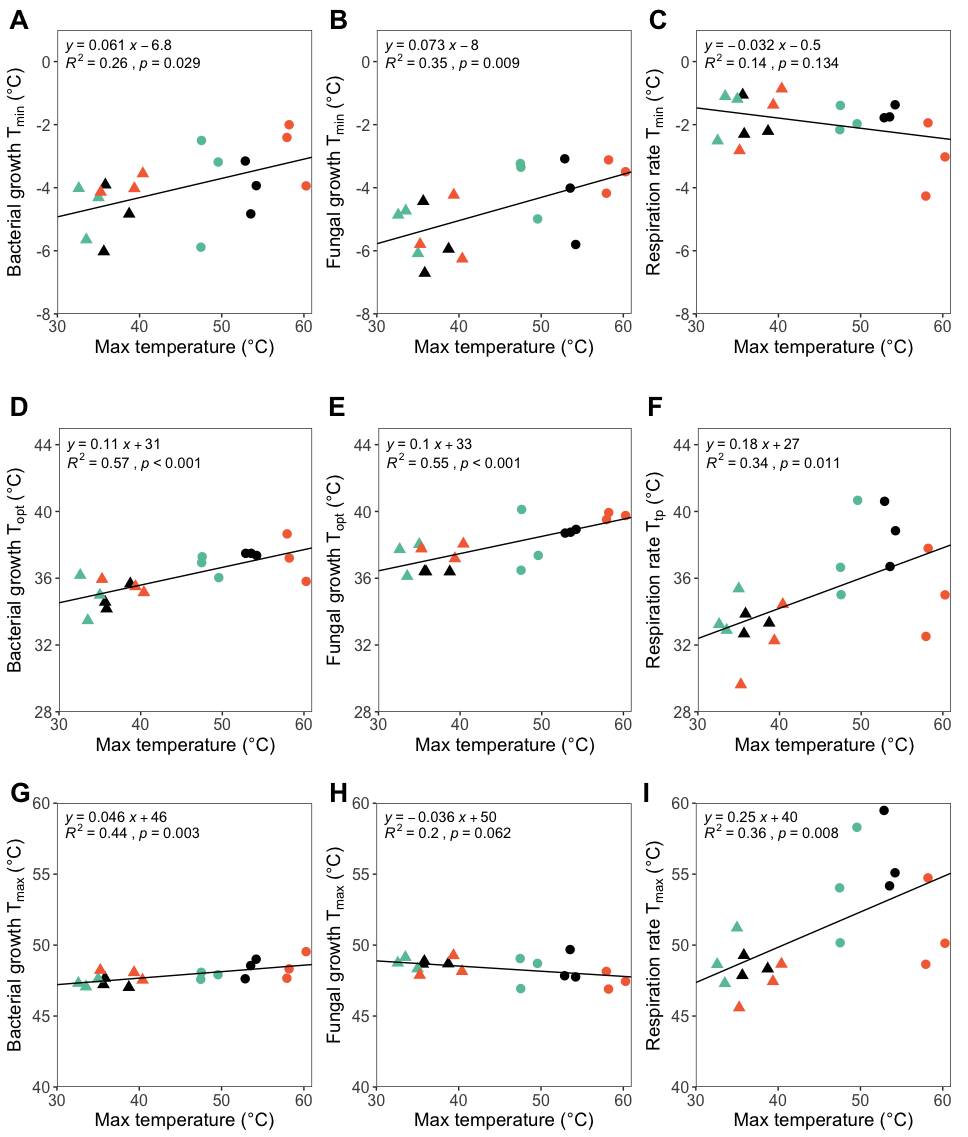
Figure S2**. Bacterial growth (A) *T_min_*, (D) *T_opt_*, (G) *T_max_*, fungal growth (B) *T_min_*, (E) *T_opt_*, (H) *T_max_* and respiration rate (C) *T_min_*, (F) *T_tp_*, (I) *T_max_*, in the different land uses (crop and forest) and treatments (colors) regressed against soil maximum temperature in each plot.

**
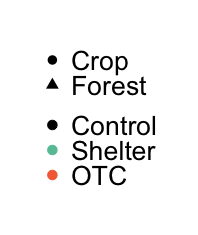

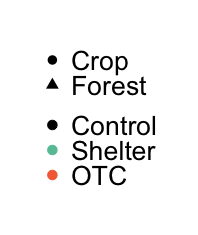
**
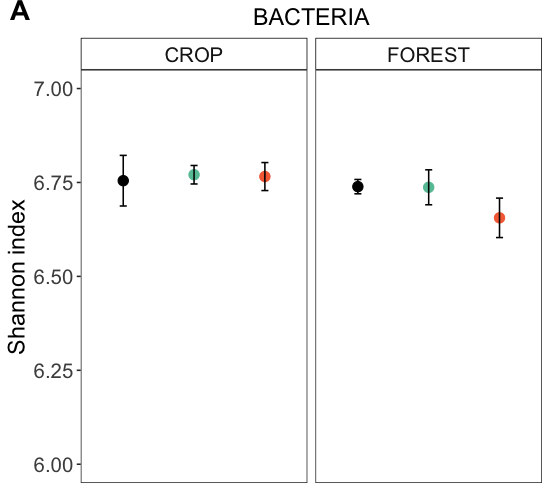
 **
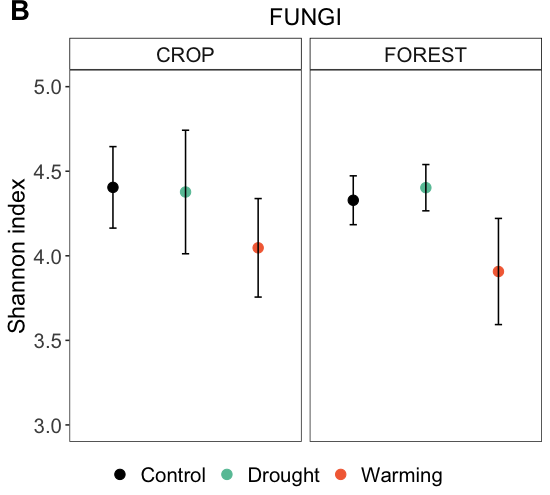
**

T: p= 0.77

L: p= 0.24

TxL: p= 0.95

T: p= 0.64

L: p= 0.20

TxL: p= 0.59

**Figure S3**. Soil microbial communities’ alpha diversity for (A) bacterial and (B) fungal communities reported as Shannon diversity index in the different land-uses (crop and forest) and treatments (colors). Values are mean values (n= 3) and errors bars represent standard errors.

**Table S4**. Correlations between environmental variables and distance matrices for bacterial and fungal communities using mantel test with Pearson correlation. Significant values are in bold.

|  | **Correlation coefficient** | **p value** |
| --- | --- | --- |
| **Bacterial community** |  |  |
| SOM | 0.92 | **0.001** |
| pH | 0.03 | 0.232 |
| maxt | 0.79 | **0.001** |
| tmin_bac | 0.18 | **0.023** |
| tmax_bac | 0.14 | **0.039** |
| topt_bac | 0.52 | **0.001** |
| MAT | 0.94 | **0.001** |
| **Fungal community** |  |  |
| SOM | 0.83 | **0.001** |
| pH | 0.19 | **0.017** |
| maxt | 0.79 | **0.001** |
| tmin_fun | 0.30 | **0.006** |
| tmax_fun | 0.21 | **0.016** |
| topt_fun | 0.40 | **0.001** |
| MAT | 0.91 | **0.001** |

**
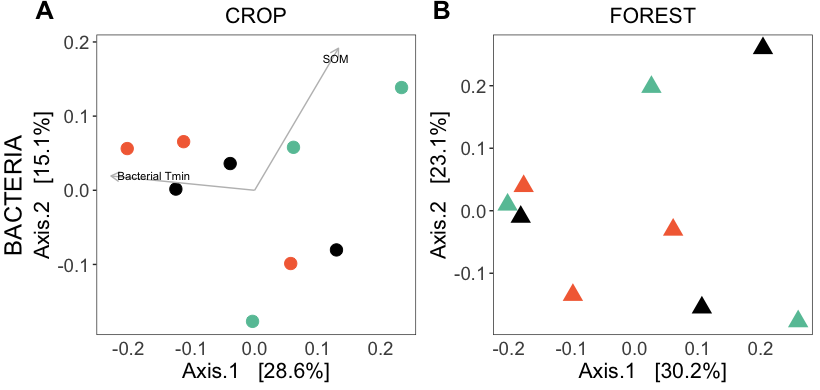
**

**
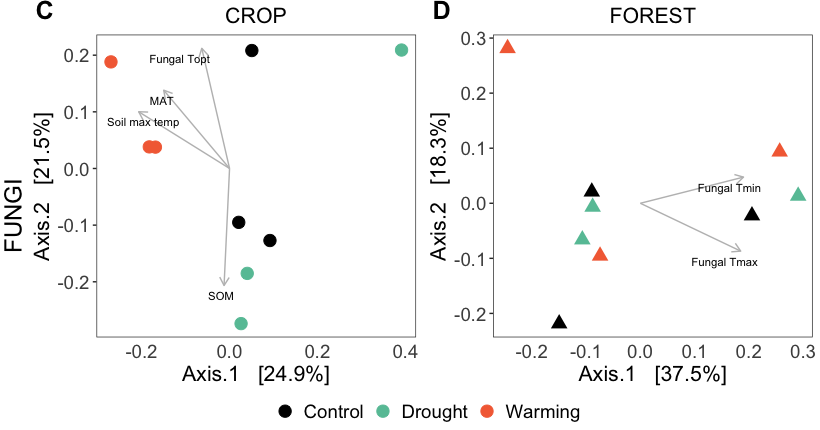
**

• Control • Shelter • OTC

**Figure S4**. PCoA plots based on Bray-Curtis dissimilarities of the bacterial (A, B) and fungal (C, D) communities separated by land-uses, crop (A, C) and forest (B, D) to reveal possible separations between treatments (colors). Arrows represent variables that correlate significantly (p<0.05) with the variance in the communities’ composition calculated with the function *envfit* from the vegan package, the length of the arrow represents the R^2^ value.

*p = 0.92*

*p = 0.98*

*p = 0.80*

*p = 0.13*

*p = 0.91*

*p = 0.43*

**C**

**B**

**A**

**
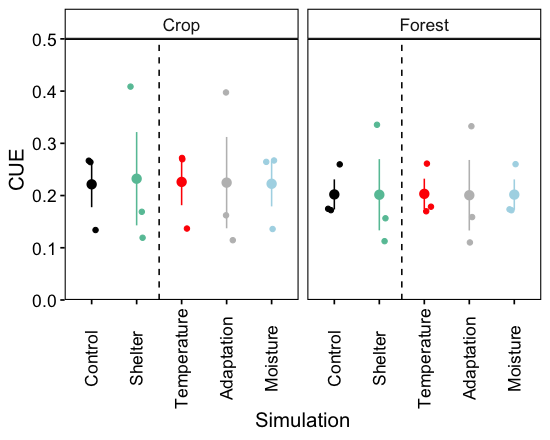

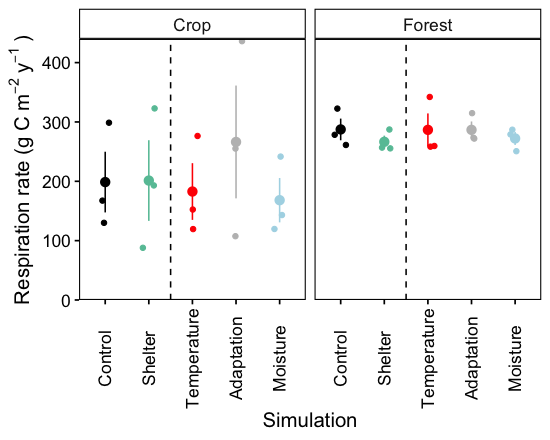

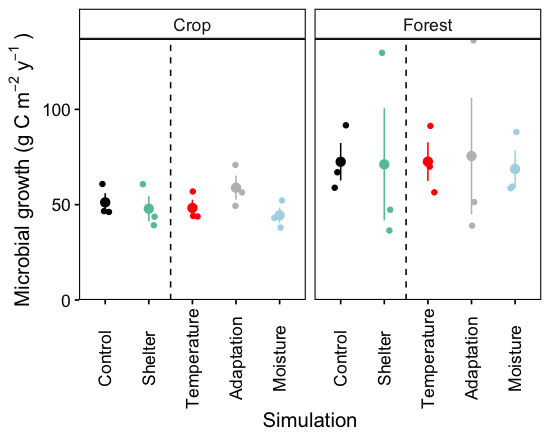
**

**Figure S5**. Simulations of cumulative (A) microbial growth, (B) respiration, and (C) carbon use efficiency in the different land-uses (crop and forest). Values are mean values (n= 3) and errors bars represent standard errors. Dots to the right of the dashed lines show the separated effect of temperature, microbial adaptation, and moisture in the shelter treatment. The p-values indicate the significance of the differences between simulations.
